# Comparative Proteomic Analysis of Flag Leaves Reveals New Insight into Wheat Heat Adaptation

**DOI:** 10.3389/fpls.2017.01086

**Published:** 2017-06-20

**Authors:** Yunze Lu, Ruiqiong Li, Ruochen Wang, Xiaoming Wang, Weijun Zheng, Qixin Sun, Shaoming Tong, Shaojun Dai, Shengbao Xu

**Affiliations:** ^1^State Key Laboratory of Crop Stress Biology for Arid Areas, College of Agronomy, Northwest A&F UniversityYangling, China; ^2^Department of Plant Genetics and Breeding, China Agricultural UniversityBeijing, China; ^3^College of Life Sciences, Liaoning Normal UniversityDalian, China; ^4^Development Center of Plant Germplasm Resources, College of Life and Environmental Sciences, Shanghai Normal UniversityShanghai, China

**Keywords:** wheat, iTRAQ, heat, photosynthesis, redox regulation

## Abstract

Hexaploid wheat (*Triticum aestivum* L.) is an important food crop but it is vulnerable to heat. The heat-responsive proteome of wheat remains to be fully elucidated because of previous technical and genomic limitations, and this has hindered our understanding of the mechanisms of wheat heat adaptation and advances in improving thermotolerance. Here, flag leaves of wheat during grain filling stage were subjected to high daytime temperature stress, and 258 heat-responsive proteins (HRPs) were identified with iTRAQ analysis. Enrichment analysis revealed that chlorophyll synthesis, carbon fixation, protein turnover, and redox regulation were the most remarkable heat-responsive processes. The HRPs involved in chlorophyll synthesis and carbon fixation were significantly decreased, together with severe membrane damage, demonstrating the specific effects of heat on photosynthesis of wheat leaves. In addition, the decrease in chlorophyll content may result from the decrease in HRPs involved in chlorophyll precursor synthesis. Further analysis showed that the accumulated effect of heat stress played a critical role in photosynthesis reduction, suggested that improvement in heat tolerance of photosynthesis, and extending heat tolerant period would be major research targets. The significantly accumulation of GSTs and Trxs in response to heat suggested their important roles in redox regulation, and they could be the promising candidates for improving wheat thermotolerance. In summary, our results provide new insight into wheat heat adaption and provide new perspectives on thermotolerance improvement.

## Introduction

Bread wheat (*Tricticum aestivum* L.) is an important staple food for human beings, however, this crop is vulnerable to heat stress and this can lead to substantial yield loss (Lobell and Tebaldi, 2014; Tack et al., 2015; Lesk et al., 2016). During the life cycle of wheat, the grain filling stage is a critical period for yield and quality, but this stage is particularly vulnerable to heat stress that can lead to greater detrimental effects in wheat production and quality than any of the other developmental stages (Zhao et al., 2007; ur Rehman et al., 2009; Farooq et al., 2011; Pradhan and Prasad, 2015). However, the mechanisms involved in the heat response and adaption in wheat, especially at the filling stage, are far from clearly understood.

Photosynthesis converts light energy into carbohydrates, a process that is sensitive to heat stress. Accumulating evidence has shown that heat stress affects multiple processes that tightly related to photosynthesis, including thylakoid membrane arrangement and damage (Prasad et al., 2008; Narayanan et al., 2015), chlorophyll content reduction (Ristic et al., 2007; Prasad et al., 2008), and Rubisco quantity and activity decline. The deactivation of Rubisco is the primary limiting factor for photosynthesis loss (Law and Crafts-Brandner, 1999; Sharkey, 2005). Moreover, high temperature reduces the quantity of chlorophyll and hastens leaf senescence (Al-Khatib and Paulsen, 1984; Prasad et al., 2008), resulting in a shorter filling duration stage and the subsequent loss of grain weight (Zhao et al., 2007; Farooq et al., 2011).

Heat stress also triggers oxidative stress and activates the reactive oxygen species (ROS)-scavenging system (Mittler et al., 2004; Suzuki et al., 2012). Severe or prolonged heat stress reduces the expression levels and enzyme activities of several major antioxidant enzymes, such as superoxide dismutase (SOD), catalase (CAT), and ascorbate peroxidase (APX), leading to ROS accumulation and a negative impact on plant growth and development (Mittler et al., 2004; Wang et al., 2011, 2015; Narayanan et al., 2015).

To counteract the detrimental effects of heat, plants have evolved multiple adaption systems. The transcriptional reprograming of the plant heat response is chartered by (1) the induction of heat shock factors and heat shock proteins (HSPs), a typical feature of the heat response; (2) the inhibition of general protein synthesis; (3) ROS burst and significant antioxidant accumulation; and (4) anabolism inhibition and catabolism activation (Morimoto, 1998; Qin et al., 2008; Mangelsen et al., 2011; Sarkar et al., 2014; Liu et al., 2015). Proteomics analyses have also revealed that HSPs, detoxification, protein synthesis and photosynthesis process are affected by heat stress (Rollins et al., 2013; Liao et al., 2014). However, in wheat, only HSPs have been characterized by proteomic analysis under heat stress in previous studies because of limited genomic information and technical restrictions (Nguyen et al., 1994; Majoul et al., 2003, 2004; Wang et al., 2015).

With updated genomic information and improvement in proteomic technology, more precise, larger-scale protein characterization in wheat has become practicable (Choulet et al., 2014; IWSGC, 2014). In this study, an isobaric tags for relative and absolute quantitation (iTRAQ)-based proteomics approach was used to analyze proteomic changes in wheat flag leaves under heat treatment. We identified 258 heat-responsive proteins (HRPs) in wheat flag leaves that were involved in several biological pathways. We also presented a detailed landscape of the reduction in photosynthesis caused by heat stress. These results enhanced our understanding of the wheat response and adaption to heat stress and provided new perspectives for improving wheat thermotolerance.

## Materials and methods

### Plant materials and growth conditions

Wheat plants (*T. aestivum* cv. Chinese Spring) were grown in a greenhouse and watered daily to avoid drought stress. The main stem ears were labeled when flowering. At 12 days after anthesis, the plants were transferred to growth chambers with a temperature regime of 24/17°C (14/10 h) day/night cycle. At 15 days after anthesis, half of the plants were applied a temperature regime of 37/17°C for 3 days and were harvested on the 4th day after 4 h under heat with light; the remaining plants were kept at 24/17°C as a control and were harvested at the same sampling time. For the experiments at the seedling stage, 10-day-old seedlings were subjected to the same conditions described above except that a different daytime temperature was used. All obtained materials were stored at −80°C for further analysis.

### Measurement of the chlorophyll and thiobarbituric acid reactive substances (TBARS) content of flag leaves and 3,3′-diaminobenzidine (DAB) staining

The chlorophyll content of flag leaves was measured according to the method of Wang et al. (2011), and the TBARS content was determined according to Lee et al. (2007). The middle part of leaf was used to determine the H_2_O_2_ content by DAB staining, as described by Tian et al. (2013). All of these determinations were performed on three biological replicates.

### Protein preparation

About 0.5 g of flag leaf was ground into a fine powder in liquid nitrogen, transferred to a 5-mL Eppendorf tube, and sonicated in lysis buffer (8 M urea, 2 mM EDTA, 1% Triton-100, 65 mM DTT, and 1% protease inhibitor cocktail). After centrifugation (20,000 g, 4°C, 10 min), the debris was removed and the protein was precipitated with cold 15% trichloroacetic acid for 2 h at −20°C. After centrifugation, the pellet was washed with cold acetone three times. The protein was resuspended in buffer (8 M urea, 100 mM triethylammonium bicarbonate, pH 8.0) and the protein concentration was determined using the 2-D Quant kit (GE Healthcare) following the manufacturer's protocol. Three independent biological replicates were performed for each sample.

### Trypsin digestion and iTRAQ labeling

Approximately 100 μg protein of each biological replicate was used for digestion. Firstly, the protein sample was reduced by the addition of 10 mM DTT for 1 h at 37°C, and then alkylated using 20 mM iodoacetamide for 45 min at room temperature in the dark. Subsequently, the protein sample was diluted with 0.1 M triethylammonium bicarbonate to reduce the urea concentration to <2 M. Then, trypsin (Promega) was added at a trypsin/protein ratio (w/w) of 1:50 for the first digestion overnight at 37°C, and the trypsin/protein ratio was adjusted to 1:100 for another 4 h digestion at 37°C to ensure the fully digestion of proteins. The tryptic peptides were desalted through a Strata X C18 SPE column (Phenomenex) and vacuum-dried, and then reconstituted in 500 mM triethylammonium bicarbonate and processed following the instructions of the 8-plex iTRAQ kit (Applied Biosystems). Briefly, one unit of the iTRAQ reagent (defined as the amount of reagent required to label 100 μg of protein) was thawed and reconstituted in 24 μl acetonitrile. The proteins of heat-stressed samples were labeled with 113, 114, and 116, and the proteins of control samples were labeled with 117, 119, and 121. The peptide mixtures of different groups were labeled differently by incubation for 2 h at room temperature and were then pooled, desalted, and dried by vacuum centrifugation.

### HPLC fractionation

The peptides were fractionated by high pH reverse-phase HPLC using an Agilent 300Extend C18 column (5 μm particles, 4.6 mm ID, 250 mm length). Peptides were separated by a gradient of 2–60% acetonitrile in 10 mM ammonium bicarbonate, pH 10, over 80 min into 80 fractions. Then, the peptides were combined into 18 fractions and dried by vacuum centrifugation.

### LC-MS/MS analysis

The collected fractions dissolved in 0.1% formic acid were separated on an EASY-nLC 1000 UPLC system. Firstly, the reconstituted samples were applied to a reversed-phase pre-column (Acclaim PepMap 100, Thermo Fisher Scientific), then separated on a reversed-phase analytical column (Acclaim PepMap RSLC, Thermo Fisher Scientific) with a gradient at a constant flow rate of 300 nL/min. The gradient was as follows: 7–20% solvent B (0.1% formic acid in 98% acetonitrile; 24 min), 20–35% solvent B (8 min), 35–80% solvent B (3 min), and 80% solvent B (4 min).

The resulting peptides were subjected to nanospray source and analyzed by tandem mass spectrometry (MS/MS) in Q Exactive™ plus (Thermo Fisher Scientific) coupled online to the UPLC system. A resolution of 70,000 in the orbitrap was used to detect intact peptides, and peptides were selected for MS/MS using 33% normalized collision energy. MS data was obtained using a data-dependent “top 20” procedure, which alternatively chooses the most abundant precursor ions with a threshold ion count above 2E4 in the MS survey scan (350–1,800 m/z). The dynamic exclusion duration was 10 s. A resolution of 17,500 was set for ion fragments detection. The electrospray voltage applied was 2.0 kV. Automatic gain control was applied to prevent overfilling of the Orbitrap; 5E4 ions were accumulated to generate MS/MS spectra. The fixed first mass was adjusted to 100 m/z.

### Database searching

The resulting MS/MS data were processed by Proteome Discoverer (Thermo scientific™ software) with Mascot search engine (v.2.3.0) and searched against UniprotTriticum_aestivum_4565.fasta (100,981 sequences in total; downloaded from Uniprot on June 5, 2015). Trypsin/P was selected as the enzyme with two maximum missing cleavages. Mass error of precursor ions and fragment ions was set to 10 ppm and 0.02 Da, respectively. Carbamidomethyl on Cys, iTRAQ-8plex (N-term) and iTRAQ-8plex (K) were specified as fixed modification and oxidation on Met was specified as variable modifications. False discovery rate was adjusted to ≤ 1%. The peptides with score ≥20 were kept for protein identification. Due to the complexity and redundancy of wheat genome, a rigorous standards were applied in protein identification, include (1) Mascot score ≥40; (2) number of unique peptides ≥2. To obtain reliable protein quantification, only the proteins that could be quantified at all six samples were assigned as quantitative proteins. To designate the HRPs, a Student's *t*-test *P* < 0.05 and fold change > 1.3 or < 0.77 across the heat-stressed and control samples were applied (Yentrapalli et al., 2013; Trevisan et al., 2015). The mass spectrometry proteomics data had been deposited to the ProteomeXchange Consortium via the PRIDE (Vizcaino et al., 2014, 2016) partner repository with the dataset identifier PXD006207 and doi: 10.6019/PXD006207.

### Bioinformatics analysis

The UniProt-GOA database (http://www.ebi.ac.uk/GOA/) and the InterProScan software (http://www.ebi.ac.uk/interpro/, v5.13-52.0) were used for gene ontology (GO) annotation. The unannotated HRPs were searched against the NCBI database further. After annotation, proteins were mapped to the pathways in Kyoto Encyclopedia of Genes and Genomes (KEGG) database (http://www.kegg.jp/). The InterPro database (http://www.ebi.ac.uk/interpro/) were used for domain annotation. Subcellular localizations of proteins were predicted by Wolfpsort (Horton et al., 2007).

### Enrichment analysis

Firstly, the number of (a) HRPs and (b) all the quantitative proteins in each domain, GO biological process and KEGG pathway term were calculated. Then the total number of (c) HRPs and (d) all the quantitative proteins annotated by domain (or the GO biological process/KEGG pathway) were calculated. Finally, these four numbers (a, b, c, d) were used to calculate Fisher's exact test *P*-values.

### Quantitative real-time PCR (qRT-PCR) analysis

Total RNA was extracted from 50 mg of leaf sample using the TRNzol reagent (TianGen, Beijing, China) following the manufacturer's protocol. About 1 μg RNA was reverse transcribed to cDNA using the PrimeScript™ RT reagent Kit with gDNA Eraser (Perfect Real Time) kit (Takara, Dalian, China) according to the kit protocol. qRT-PCR was performed on an Applied Biosystems 7300 Real-Time PCR System using the SYBR® Premix Ex Taq™ II (Tli RNaseH Plus) kit (Takara, Dalian, China) following the corresponding protocol.

## Results

### Proteome profiles of wheat flag leaves under heat stress

**Figure 1:**
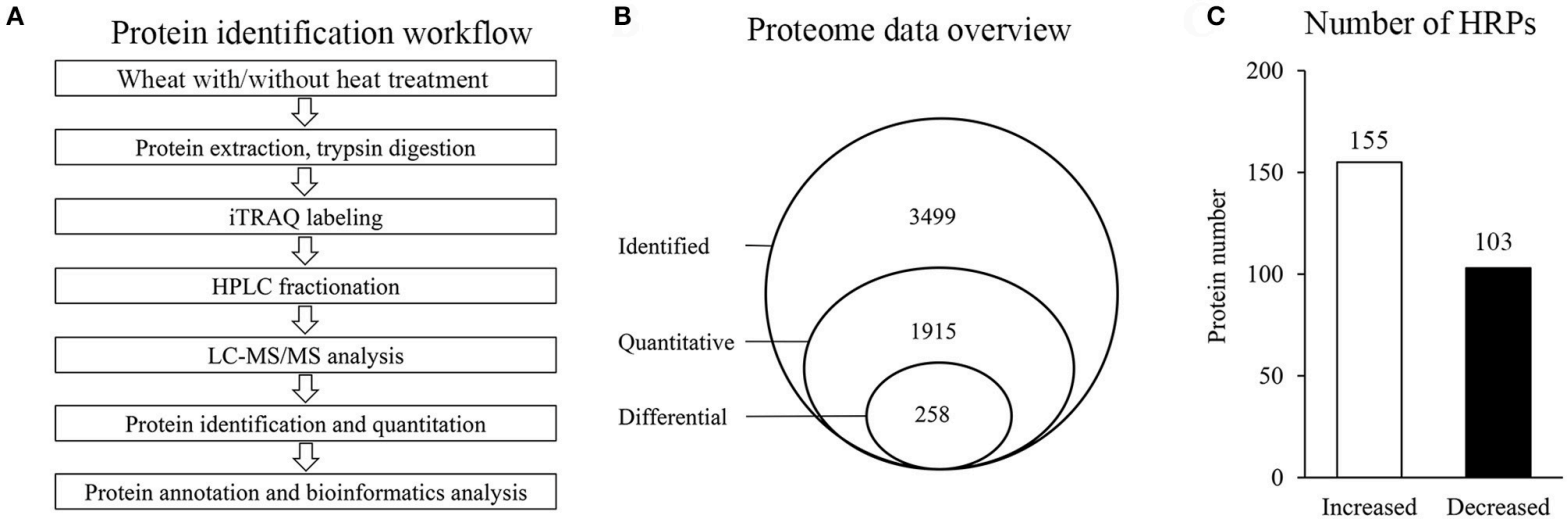
Proteins identification in this experiment. **(A)** General workflow of the experiment for iTRAQ-based proteome analysis of wheat with/without heat stress. **(B)** Schematic diagram of proteins characterization. **(C)** Number of HRPs.

To investigate the proteome alterations of wheat flag leaves under heat stress, iTRAQ-based quantitative proteomics analysis was conducted (Figure 1A). Among the 3499 proteins identified, 1915 could be further quantified (Supplementary Tables 1, 2), and 258 were assigned as HRPs (Figure 1B), including 155 heat-increased proteins and 103 heat-decreased proteins (Figure 1C).

**Figure 2:**
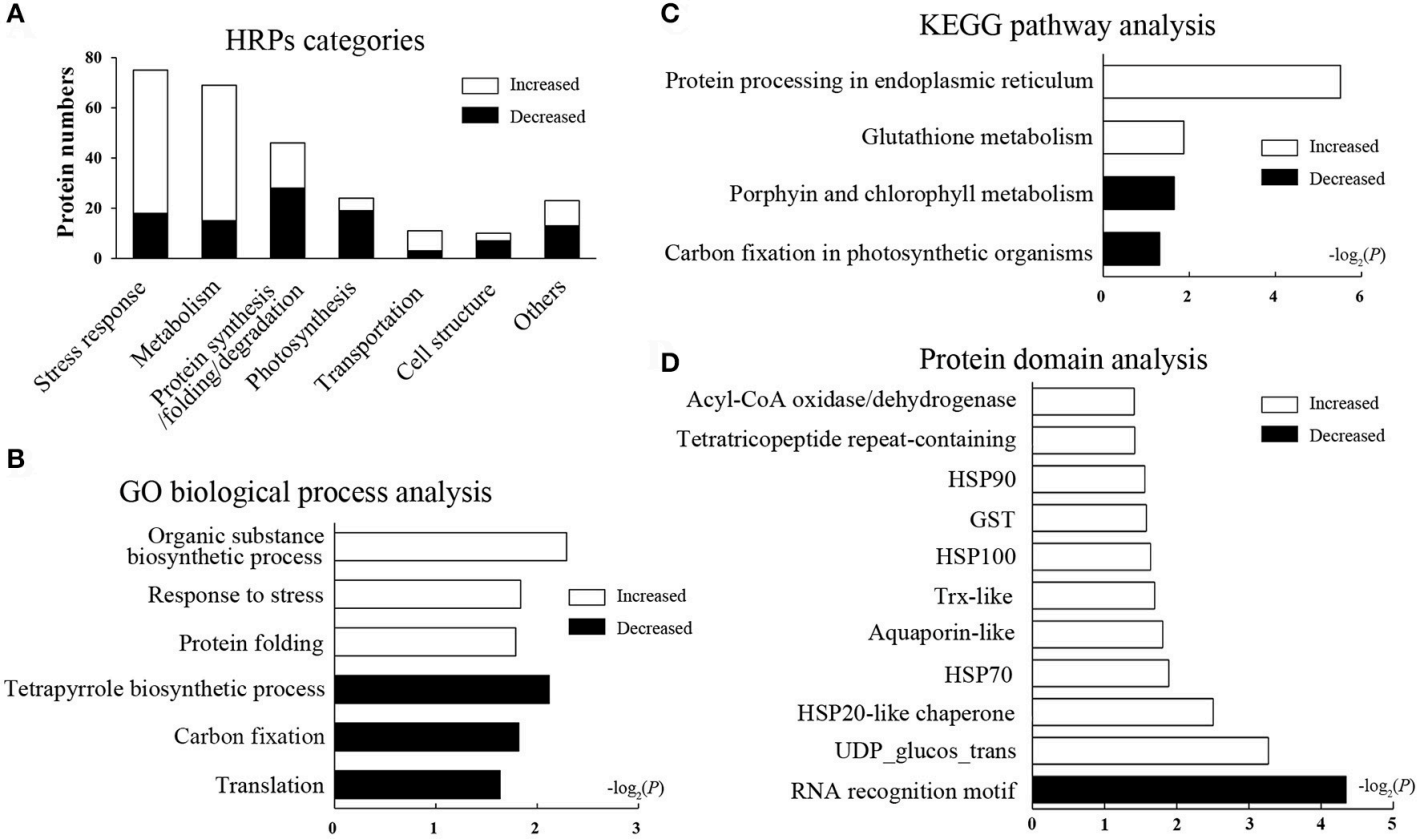
Function categories and enrichment analysis of HRPs. Black bars represent decreased proteins, white bars represent increased proteins. **(A)** Functional classification of HRPs. **(B)** GO biological process analysis. The bars represent −log_2_(*P*) where *P* represents the Fisher' exact test *P*-values, and so as it in **(C,D)**. **(C)** KEGG pathway analysis. **(D)** Domain enrichment analysis.

The HRPs were classified into seven categories according to the GO database and the Uniprot knowledgebase (Figure 2A, Supplementary Table 3, including stress response (29.1%), metabolism (26.7%), protein synthesis/folding/degradation (17.8%), photosynthesis (9.3%), and other categories. The categories affected suggest that heat stress has an effect on diverse biological processes in wheat leaves.

### Enrichment analysis of HRPs

To illustrate the major heat-responsive processes in wheat flag leaves, enrichment analysis was performed on the GO biological process, the KEGG pathway and the protein domain (Supplementary Table 4). Among the biological process, as expected, the GO term “response to stress,” and “protein folding” were significantly enriched in increased HRPs. Whereas, “tetrapyrrole biosynthetic processes,” “carbon fixation,” and “translation” were remarkably enriched in decreased HRPs (Figure 2B). These results indicated that the stress response and protein folding were activated, whereas translation and photosynthesis were impeded by heat stress.

In the pathway enrichment analysis, protein processing in endoplasmic reticulum (ER) and glutathione metabolism were significantly enriched, and most proteins in these pathways were heat increased. Whereas, porphyrin and chlorophyll metabolism, and carbon fixation in photosynthetic organisms were enriched, and most related proteins were decreased in heat conditions (Figure 2C). These results further suggested that protein processing and photosynthesis were remarkably affected by heat stress accompanied by redox system alterations.

In the domain enrichment analysis, HSPs (including HSP100, HSP90, HSP70, and HSP20), the glutathione-S-transferase (GST) domain, the thioredoxin (Trx)-like fold, the tetratricopeptide repeat-containing domain, and others were remarkably enriched as heat-induced domains (Figure 2D), indicating these domains may be the central heat responsive domains. Whereas, only the RNA recognition motif domain was enriched as a heat-suppressive domain. The domain enrichment analysis revealed that the stress response, protein folding, and redox regulation were all activated by the heat stress in flag leaves.

Taken together, the enrichment analyses based on the proteomics data suggested that protein synthesis and folding, redox regulation, and photosynthesis were the most heat-responsive processes.

### HRPs in protein synthesis and folding

**Figure 3:**
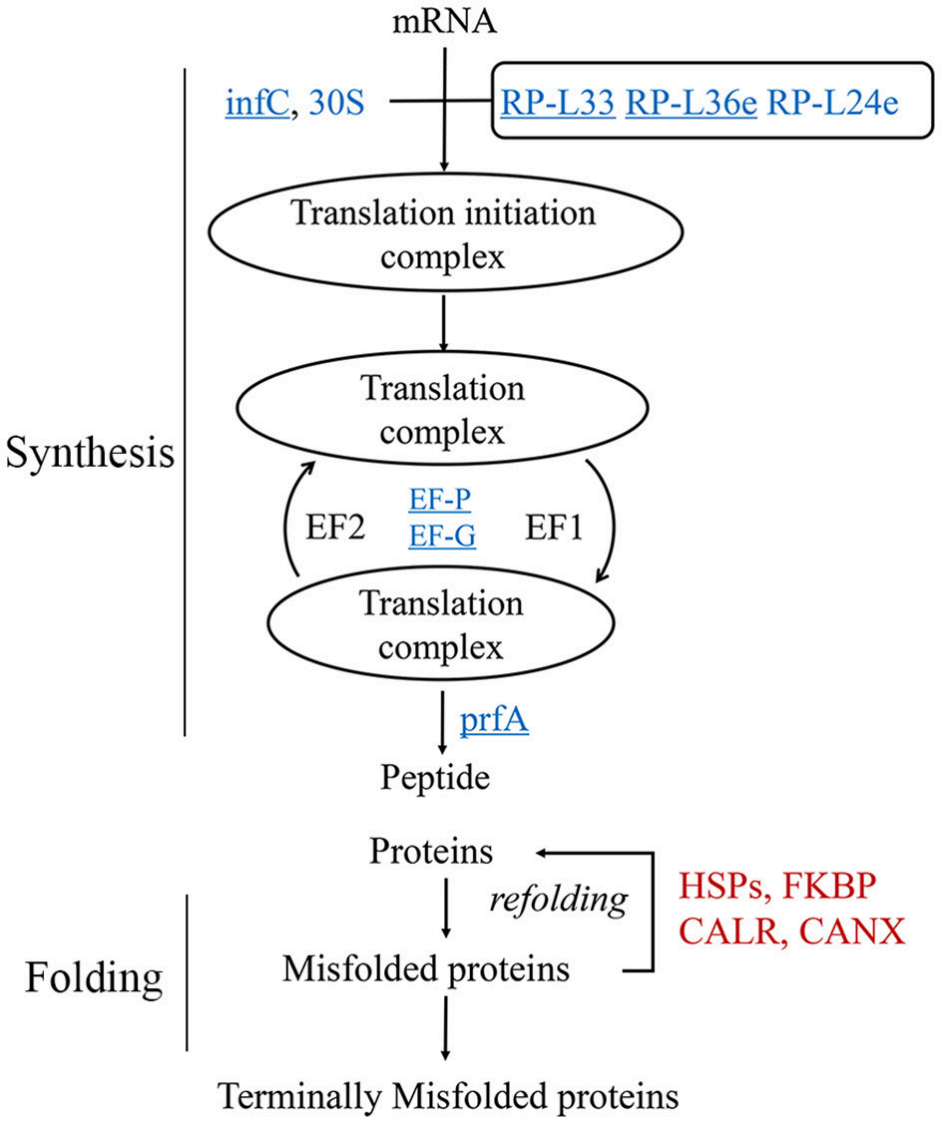
Regulation of protein synthesis and turn over activity. Protein names in blue were decreased, while red ones were increased. The underlined proteins were present in the enriched “translation” biological process in Figure 2B. 30S, 30S ribosomal protein; infC, translation initiation factor IF-3; EF-G, elongation factor G; EF-P, elongation factor P; RP-L, large subunit ribosomal protein; prfA, peptide chain release factor 1; CALR, calreticulin; CANX, calnexin; FKBP, FK506-binding protein.

The proteomics data suggested that protein translation was decreased during heat stress (Figure 2B). To clarify the translational changes in wheat leaves under heat stress, *de novo* protein synthesis was evaluated based on HRPs (Figure 3). The translation components (translation initiation factor IF-3, elongation factors P and G, peptide chain release factor 1, ribosomal proteins) showed decreased under heat stress. These results indicated that translation elongation was coincidentally interfered by heat, in addition to the previously reported heat-inhibition of translation initiation (Spriggs et al., 2010; de Nadal et al., 2011).

However, the chaperones involved in protein folding were remarkably heat induced (Figure 2B), especially the HSPs (Figure 2D). In this study, 26 of the 29 heat-responsive HSPs were accumulated, suggesting that their critical roles in heat response in wheat leaves. Other chaperones, for example, calnexin and calreticulin were coincident heat accumulated. A set of proteolytic enzymes were also identified, the cysteine proteinase RD21a and serine carboxypeptidase-like clade I were coincident accumulated, while subtilisin-like protease were differentially regulated.

HRPs involved in protein metabolism suggested that wheat leaves preferentially protected existing proteins by chaperones, instead of *de novo* substrates synthesis in response to heat stress.

### GSTs and Trxs were significantly accumulated in response to heat stress

**Figure 4:**
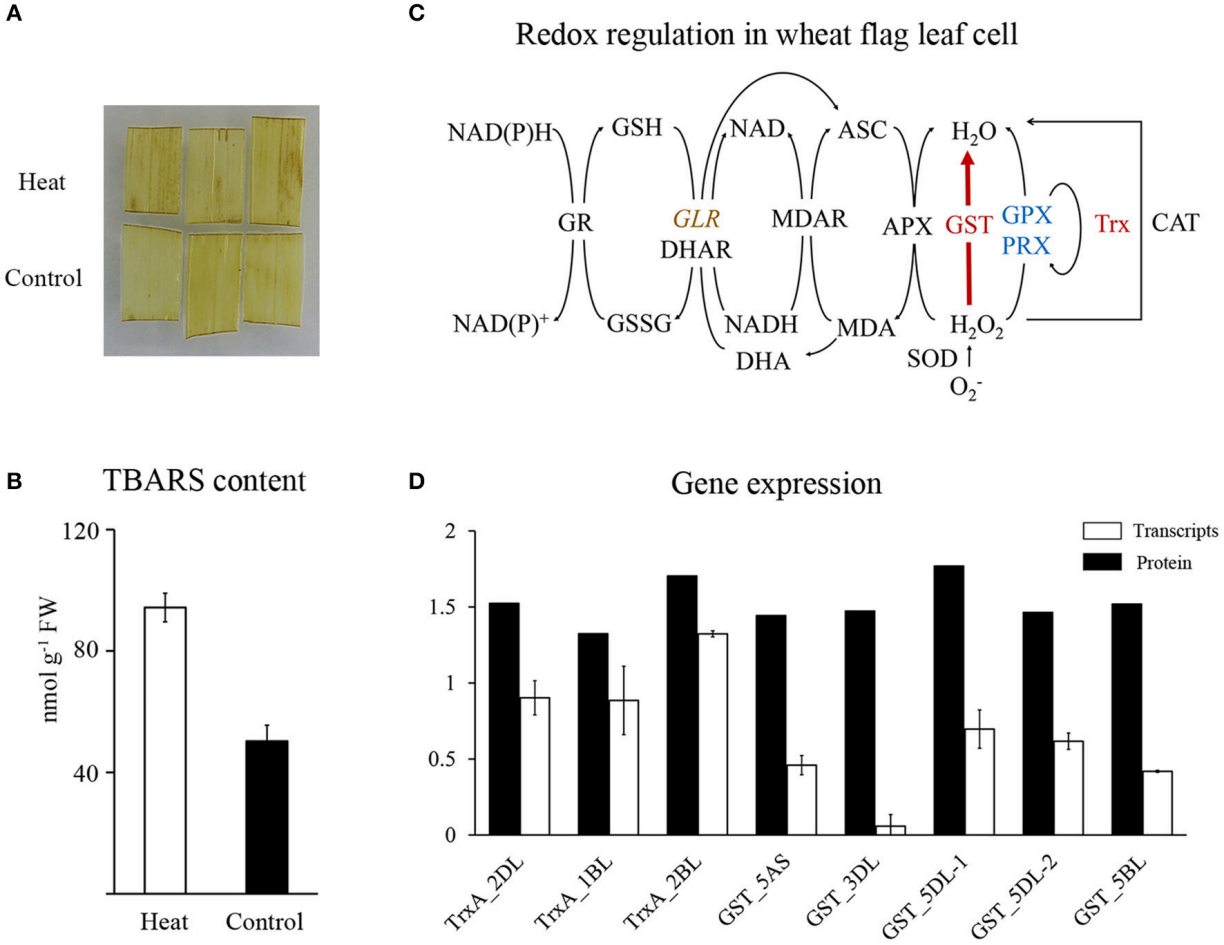
Redox regulation in wheat flag leaf in heat condition. **(A)** DAB staining of wheat flag leaves. **(B)** TBARS content of wheat flag leaves. **(C)** Schematic representation of redox regulation. Red represent increased, blue represent decreased, brown with italic represent differentially regulated. Map was drawn based on Mittler et al. (2004). APX, Ascorbate peroxidase; ASC, Ascorbate; CAT, Catalase; DHA, Dehydroascorbate; DHAR, Dehydroascorbate reductase; GLR, Glutaredoxin; GPX, Glutathione peroxidase; GR, Glutathione reductase; GSSG, glutathione disulphide; GST, Glutathione S-transferase; MDAR, Monodehydroascorbate reductase; Trx, Thioredoxin; Prx, Peroxiredoxin; SOD, Superoxide dismutase; **(D)** qRT-PCR analysis of Trxs and GSTs.

The glutathione-mediated redox system was significantly enriched in HRPs (Figure 2C), suggesting a remarkable alteration in the redox status of cells under heat stress. However, DAB staining showed that the H_2_O_2_ content was slightly affected by the applied heat stress (Figure 4A). Then, the TBARS content, which predicts the peroxidation and destruction of lipids with subsequent membrane damage caused by ROS (Liu et al., 2014), showed significant accumulation (Figure 4B), indicating that membrane damage was induced by the oxidative stress caused by heat.

To reveal the major players that maintained redox homeostasis in wheat leaves, heat-responsive antioxidant proteins were investigated and mapped to the redox regulation system (Figure 4C). Twelve GSTs and three Trxs showed significant accumulation (Figure 2D), with increase of polyphenol oxidase and decrease of three other redox regulatory enzymes (glutaredoxin, glutathione peroxidase, and peroxiredoxin). Our proteomics data suggested that GSTs and Trxs were the major players in regulating redox alterations in heat-stressed wheat leaves.

Unexpectedly, in contrast to the accumulation of proteins, the transcripts of GSTs and Trxs in the same heat-stressed samples were reduced or remained relatively stable, respectively Figure 4D, Supplementary Table 5. These results suggested that the significant increases in GSTs and Trxs may result from post-transcriptional regulation of long-term heat stress.

### Carbon fixation and chlorophyllide synthesis were remarkably affected by heat stress

**Figure 5:**
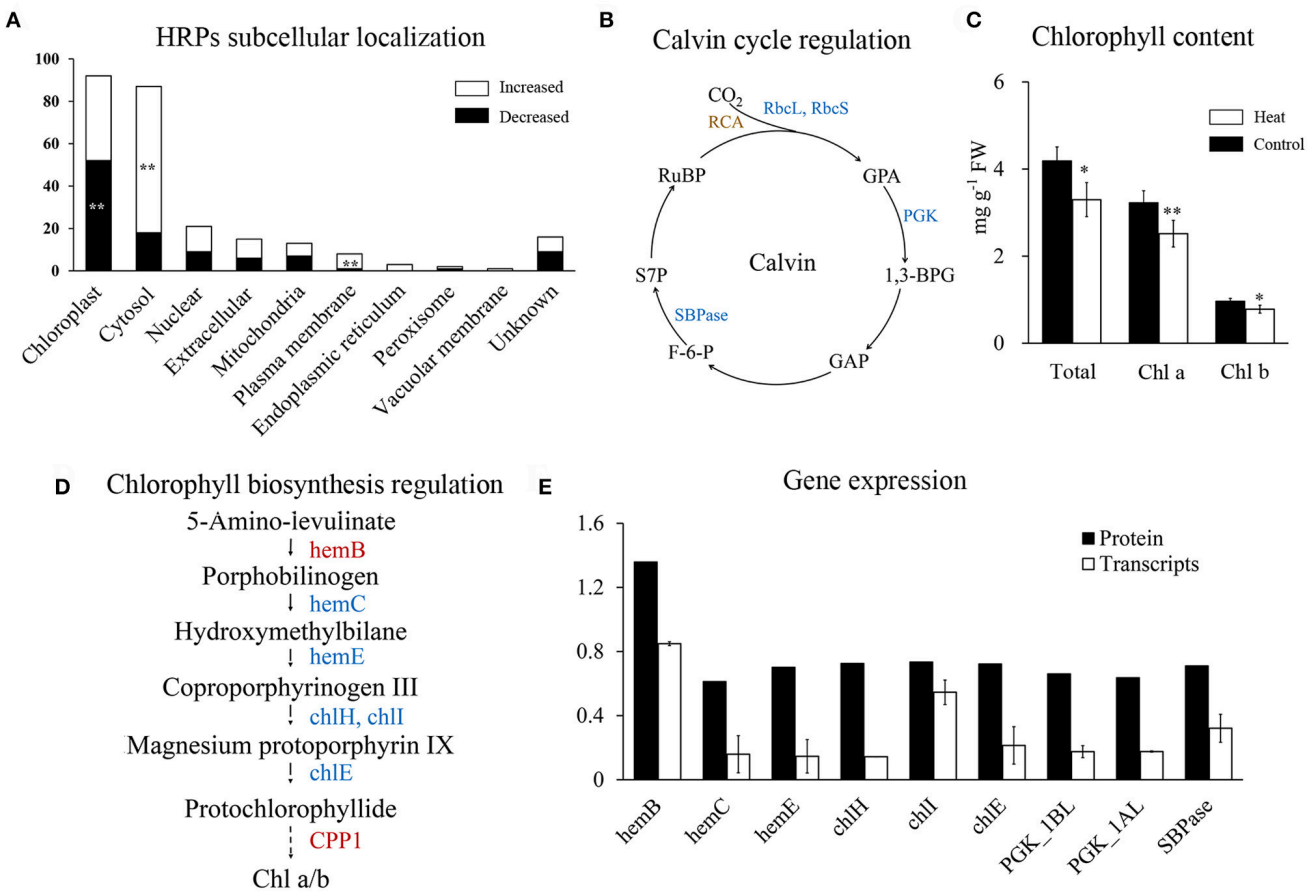
Regulation of photosynthesis in wheat leaf under heat stress. **(A)** Subcellular localization of HRPs predicated by Wolfpsort. White bars represent increased proteins, black bars represent decreased proteins. **(B)** Map of Calvin cycle regulation. Blue represent decreased, brown represent differentially regulated. 1,3-BPG, 1,3-Bisphospho-D-glycerate; F-6-P, D-Fructose 6-phosphate; GAP, D-Glyceraldehyde 3-phosphate; GPA, D-Glycerate 3-phosphate; PGK, phosphoglycerate kinase; RCA, Rubisco activase; rbcL, Rubisco large chain, rbcS; Rubisco small chain; RuBP, D-Ribulose 1,5-bisphosphate; S7P, D-Sedoheptulose 7-phosphate; SBPase, sedoheptulose-bisphosphatase; **(C)** Chlorophyll content of wheat flag leaves. Asterisk represent significantly different at *p* < 0.05 level, double asterisks at 0.01 level. **(D)** Map of chlorophyll biosynthesis regulation. Red represent increased, blue represent decreased. chlE, magnesium-protoporphyrin IX monomethyl ester (oxidative) cyclase; chlH, magnesium chelatase subunit H; chlI, magnesium chelatase subunit I; hemB, porphobilinogen synthase; hemC, hydroxymethylbilane synthase; hemE, uroporphyrinogen decarboxylase **(E)** qRT-PCR analysis of genes in chlorophyll synthesis and Calvin cycle.

In this study, HRPs in chloroplasts showed significantly decrease under heat stress (Figure 5A), which indicated that processes in chloroplast maybe repressed. Photosynthesis is an already known heat-sensitive process (Farooq et al., 2011; Prasad et al., 2011), correspondingly, 19 out of the 24 photosynthesis-related HRPs involved in chlorophyll synthesis, photosynthetic electron transport, and the Calvin cycle, showed a decrease in abundance after heat stress. Furthermore, porphyrin and chlorophyll metabolism, and carbon fixation were significantly enriched among the decreased HRPs (Figure 2C). These results indicated photosynthesis was affected through multiple processes during heat stress.

Rubisco is crucial for carboxylation in the Calvin cycle and it is known to be inhibited by heat stress (Lee et al., 2007; Wang et al., 2015). In our results, all the four heat-responsive Rubisco large subunits and Rubisco small subunits were significantly decreased (Supplementary Table 4). In addition, chloroplast phosphoglycerate kinase and sedoheptulose-bisphosphatase were also decreased (Figure 5B), indicating that heat stress has a negative impact on the Calvin cycle.

Chlorophyll metabolism was significantly enriched in decreased HRPs (Figure 2C), indicating that the chlorophyll content of flag leaves may be reduced during heat stress. Chlorophyll quantification confirmed that the chlorophyll content decreased with heat stress (Figure 5C). Furthermore, both chlorophyll a and b were decreased (Figure 5C), indicating that their common precursor was likely reduced during heat stress. Correspondingly, five HRPs (hemE, hemC, chlE, chlH, and chlI) that were critical for the synthesis of protochlorophyllide from porphobilinogen were significantly decreased (Figure 5D). This finding indicated that the inhibition of protochlorophyllide biosynthesis was likely due to the decreased abundance of these proteins.

Further, qRT-PCR quantification showed that the mRNAs of all of the HRPs involved in chlorophyll synthesis and carbon fixation were significantly downregulated under heat stress (Figure 5E). Changes in the mRNA levels were more severe than those at the protein level, indicating that the decreases in chlorophyll synthesis and carbon fixation may be initially regulated at the transcriptional level during heat stress.

### Expression pattern of photosynthesis-related genes during continuous heat stress

**Figure 6:**
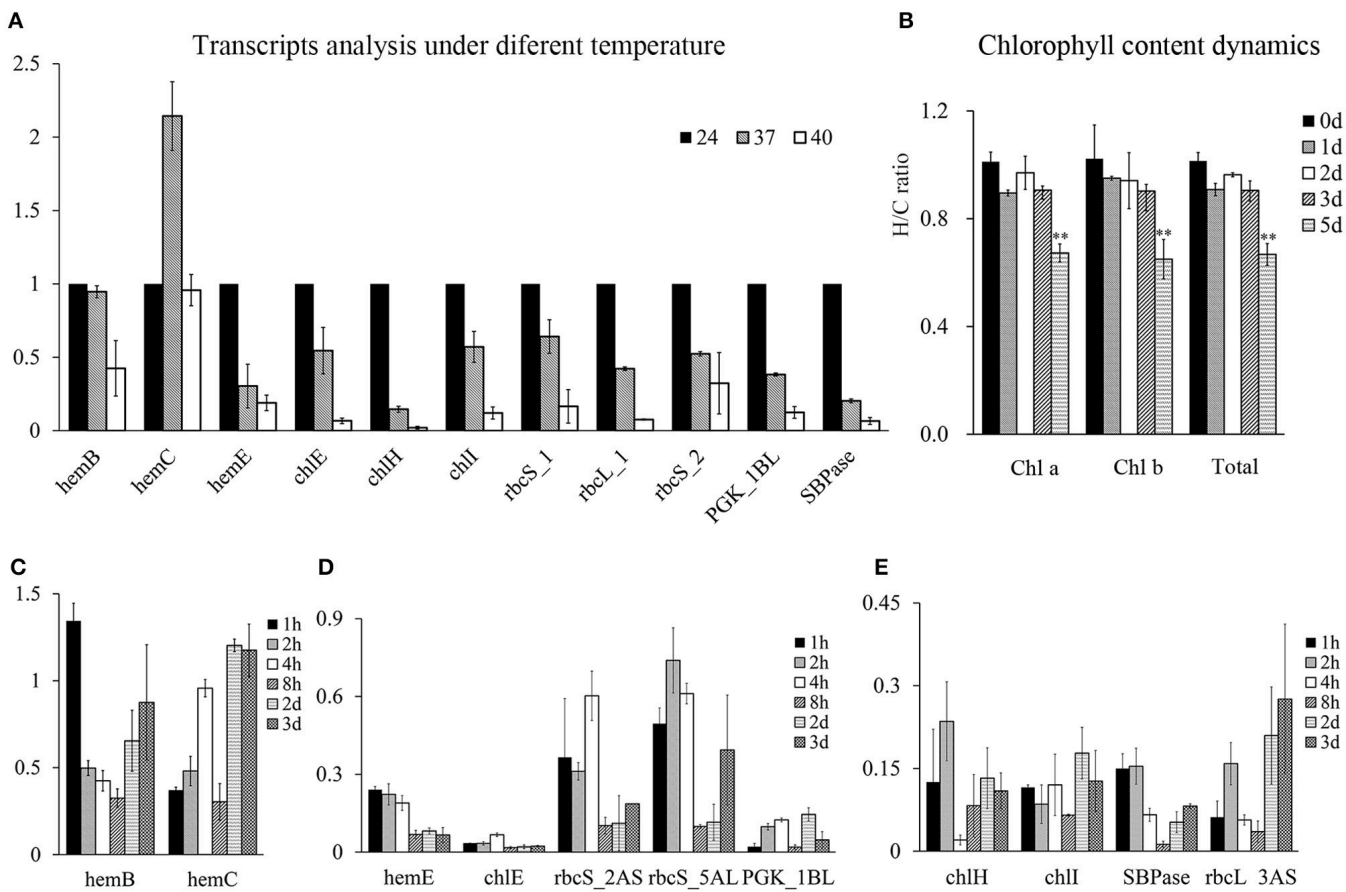
Chlorophyll content dynamics and the photosynthesis process related genes expressions under heat stress at wheat seedling stage. The Y-axis represent fold changes (heat to control) in all figures. **(A)** Transcripts analysis of genes involved in chlorophyll synthesis and Calvin cycle under 24, 37, 40°C for 4 h under light condition. **(B)** Chlorophyll content dynamics under daylight heat stress at 40°C. **(C–E)**, time course analysis of mRNAs of chlorophyll synthesis and Calvin cycle related genes. Samples at time point of 2 and 3 d were obtained 4 h after light with heat stress.

The results above demonstrated that HRPs involved in photosynthesis were all encoded by heat sensitive genes. To illustrate their heat response patterns, transcripts analysis were conducted at wheat seedling stage and showed that most of them were inhibited by temperature rises (Figure 6A), suggesting that the process of photosynthesis was rapidly regulated at the transcriptional level following heat stress. However, the rapid downregulation of genes involved in chlorophyll precursor synthesis did not result in substantial chlorophyll loss until 4–5 days later (Figure 6B).

Therefore, the time course analysis of the mRNA of these genes under severe heat stress (40°C) was performed. The results showed that the transcripts of these proteins were encoded by short-term heat-responsive genes that were downregulated within 1–2 h following heat stress, as reported by previous transcriptome analysis (Liu et al., 2015). However, in the prolonged heat treatment, these genes displayed different transcriptional profiles. *HemB* and *HemC* were recovered at the transcriptional level, indicating a heat acclimation pattern (Figure 6C), whereas the others genes were severely heat-suppressed (Figures 6D,E), with five of the nine genes displaying a further decrease in transcription with recurring heat stress, indicating the accumulated effects of heat stress (Figure 6D).

## Discussion

### Induced chaperones are key players in wheat leaf heat adaptation

Protein folding and ER-associated degradation system are important components of ER quality control, unfold proteins stress is induced when the misfolded proteins are over aggregated when plants are suffered from environment stress (Liu and Howell, 2010; Liu and Li, 2014; Ruggiano et al., 2014). Chaperones and cochaperones protect substrates from aggregation and aid proteins refolding. Furthermore, increases in chaperones have been proposed to enhance heat tolerance in plants and maintain yields under heat stress (Jia et al., 2008; Salvucci, 2008; Chauhan et al., 2012; Zhong et al., 2013). In our results, many chaperones together with some proteasome subunits were accumulated, suggesting that protein folding and ER-associated degradation system are induced to maintain normal plant growth and development during heat stress. Further, studies to characterize substrates of these chaperones will be critical for clarifying their specific roles. In addition, due to complexity of protein quality control and heat stress response, interactions within chaperones/cochaperones and between substrates also need more efforts to illustrate their protection networks (Wang et al., 2004; Kotak et al., 2007; Hahn et al., 2011). Among these chaperones, accumulating evidence demonstrates that HSP induction is the conserved mechanism for plant adaptation, and may be considered to be a potential target for molecular-assisted selection in thermotolerance breeding.

In contrast to the significant accumulation of chaperones, the proteolytic enzymes or components were differentially regulated, possibly due to their different roles. For example, serine carboxypeptidase-like clade I were accumulated, but the clade II was decreased. In Arabidopsis, clade I may act as acyltransferases while clade II were regarded as genuine carboxypeptidases (Fraser et al., 2005), and their roles in plant growth in heat stress need further characterizations.

### GST and Trx play a crucial role in redox regulation in leaves under heat stress

As a response to the oxidative burst, several antioxidant enzymes such as CAT, SOD, and APX (Lee et al., 2007; Qin et al., 2008; Liu et al., 2014) are often induced to maintain redox homeostasis in plants. However, their abundance was not induced by heat stress in this study, as shown in previous heat stress studies (Shin et al., 2005; Lee et al., 2007; Wang et al., 2011, 2015). By contrast, GSTs and Trxs that were also involved in maintaining redox homeostasis in plants (Dixon et al., 2002; Park et al., 2009; Zhang et al., 2011; Zagorchev et al., 2013; Labrou et al., 2015), played a prominent role in redox regulation in our analysis. Accumulation of GST was thought to contribute to thermotolerance by reducing ROS production (Xu and Huang, 2008). This finding inferred an unusual redox regulatory system comprising GSTs and Trxs was activated in response to redox changes within leaves induced by heat stress.

After 3 days of heat stress, the mRNA level of GSTs and Trxs were reduced or maintained at a relatively stable level, respectively (Figure 4D). Some of these genes showed similar expression patterns to those reported in previous study (Liu et al., 2015), suggesting that the accumulation of these proteins may result from post-transcriptional regulation. The contrast between the mRNA and protein expression level of GSTs may be due to their translation efficiencies, perhaps mediated by alternative translation mechanisms such as those of HSPs (Spriggs et al., 2010). Furthermore, their interactions with HSPs (Robin et al., 2003; Montrichard et al., 2009; Raza, 2011), may confer GST and Trx proteins more thermostability (Gautier et al., 1998; Yamamoto et al., 2005, 2007; Traverso et al., 2007), and guarantee their functions in heat stress. These results suggest that GSTs and Trxs may be promising candidates for improving wheat thermotolerance.

### Inhibition of photosynthesis following heat stress

Photosynthesis is the basis for plant growth and development. Previous studies have demonstrated that heat stress significantly reduced photosynthesis (Ristic et al., 2007; Fu et al., 2008; Prasad et al., 2008; Wang et al., 2011; Narayanan et al., 2015). Our proteomics data showed that most HRPs involved in chlorophyll synthesis, photosynthetic electron transport, and the Calvin cycle were heat-suppressed, providing a global perspective for the underlying mechanism of decreased photosynthesis during heat stress. Moreover, the HRPs involved in photosynthesis that were decreased in abundance showed reduced levels of transcription, indicating that these HRPs may not be the direct heat damage targets but rather may be indirectly regulated at the transcriptional level.

A potential option for improving wheat thermotolerance would be to enable heat adaptation of photosynthesis process, which we showed to suffer the accumulated effects of heat stress. A previous study showed that short-term heat stress conferred less loss of floret fertility and individual grain weight compared with long-term heat stress, which also demonstrated a cumulative heat response (Prasad and Djanaguiraman, 2014). Thus, short episodes of heat stress may result in mild damage to wheat growth, which permits potential recovery under normal conditions. Therefore, extending the heat tolerance period may be a potential breeding target to achieve improved wheat thermotolerance.

## Author contributions

YL, SX, XW, and WZ performed proteomic data analysis. YL, RL, RW, and ST performed sample collection, mRNA quantification and physiological experiments. SX, YL, QS, and SD designed the study, and organized all the data and wrote the manuscript. All authors were involved with the revision of the manuscript and approved the final manuscript.

### Conflict of interest statement

The authors declare that the research was conducted in the absence of any commercial or financial relationships that could be construed as a potential conflict of interest.
